# Pulmonary valve endocarditis in a postpartum patient: a case report of unconventional risk

**DOI:** 10.1097/MS9.0000000000004875

**Published:** 2026-03-31

**Authors:** Bishweshwar Joshi, Dhiraj Adhikari, Bishal Budha, Neetika Paudel, Shivam Jha, Tekraj Upadhaya

**Affiliations:** Department of Internal Medicine, Maharajgunj Medical Campus, Tribhuvan University Teaching Hospital, Institute of Medicine, Kathmandu, Nepal

**Keywords:** anemia, case report, culture negative, postpartum, pulmonary valve, right-sided endocarditis

## Abstract

**Introduction:**

Infective endocarditis is a critical and fatal condition characterized by infection of the endocardium, including the heart valves, cardiac devices, central catheters, and nonfunctional embryonic remnants of the right atrium, such as the Eustachian valve. Intravenous drug users, people with intracardiac devices and central venous catheters are significantly more likely to develop right-sided endocarditis.

**Case presentation:**

A 35-year para 3 woman, on her 40th postpartum day after normal vaginal delivery at home, was referred to our center with symptoms of headache, fever, shortness of breath, palpitation manifested on the 5th postpartum day and conservatively managed in a peripheral institute until being referred. She was initially managed symptomatically but again presented with worsening symptoms, and on CECT, acute pulmonary thromboembolism with pulmonary infarcts was seen. Similarly, the echocardiographic finding showed hypoechoic oscillating structure of 32 mm × 9 mm attached to pulmonary valve (probably clot) with severe pulmonary regurgitation, mild eccentric tricuspid regurgitation, dilated right atrium and right ventricle. Hence, a diagnosis of right-sided infective endocarditis was made and the patient was managed accordingly.

**Discussion:**

This case report presents a rare instance of right-sided infective endocarditis involving the pulmonary valve in a postpartum patient without any associated risk factors. Postpartum progesterone suppression, physiological stress, and uterine healing create a temporary immunocompromised state with weeks-long infection vulnerability after delivery. Initially, the patient was misdiagnosed as a case of postpartum cardiomyopathy. The corrected diagnosis of infective endocarditis was confirmed via echocardiography despite negative blood cultures. Severe anemia (Hb 5.2 g/dl) and postpartum immune alterations likely predisposed to infection. The patient improved on empirical antibiotics (clindamycin/linezolid) and rivaroxaban. This case highlights the importance of considering right sided infective endocarditis in postpartum fever presenting with shortness of breath and the challenges of diagnosis in resource-limited settings.

**Conclusion:**

This case highlights right-sided infective endocarditis (RSIE) as a rare but serious complication in the postpartum period, even in patients without traditional risk factors. The postpartum state marked by progesterone-driven immunosuppression, hemodynamic stress, and anemia-induced immune dysfunction, may predispose women to atypical infections, including culture-negative endocarditis.

## Introduction

Infective endocarditis (IE) is a critical and potentially fatal condition characterized by infection of the endocardium, including the heart valves, cardiac devices, central catheters, and nonfunctional embryonic remnants of the right atrium, such as the Eustachian valve^[^1^]^. Without timely intervention and management, a plethora of intracardiac and far-reaching extra-cardiac complications may occur. The predominant cases of infectious endocarditis are caused by gram-positive *Streptococci, Staphylococci,* and *Enterococci*. Together, these three groups account for 80–90% of all cases. *Staphylococcus aureus* is responsible for around 30% of cases occurring in the developed world. In the immunocompromised population, fungal endocarditis, which accounts for just 1% of cases, can be a potentially lethal consequence of systemic *Candida* and *Aspergillus* infection^[^2^]^. Any patient with risk factors who presents with fever or sepsis of unknown cause should be evaluated for infective endocarditis, which can present with a wide range of symptoms such as low-grade fever (<39 °C), night sweats, fatigability, malaise, and weight loss. Chills and arthralgia may occur. Symptoms and signs of valvular insufficiency may be a first clue and physical examination findings may be normal or manifest pallor, fever, change in an already developed murmur, or new onset murmur and tachycardia^[^3,4^]^, Within the first month, patients typically notice the subtle start of fever, chills, malaise, and fatigue, which usually leads to a medical evaluation.

Infective endocarditis can have an indolent, sub-acute, or a more acute, fulminant course with greater potential for quick onset decompensation, and duration of symptoms may vary based on this classification, while the manifestations may be more or less similar^[^4^]^. Most typically, infectious endocarditis affects the left side of the heart, such as the aortic or mitral valve. The right-sided tricuspid or pulmonic valve accounts for 5–10% of instances^[^4^]^. Intravenous drug users, people with intracardiac devices and central venous catheters are significantly more likely to develop right-sided endocarditis^[^1^]^. This case-report aims to shed light on the unique case of 35-year-old woman who presented to our center from the rural part of Nepal on 40th day of her postpartum period following normal vaginal delivery at home, with symptoms of fever, chills, rigor and sweating, on and off shortness of breath, palpitation, who after meticulous evaluation was diagnosed with pulmonary valve endocarditis, despite having no risk factor for endocarditis. We have reported this rare case in line with SCARE guidelines^[^5^]^.

## Case presentation

This is a case of a 35-year-old para 3 woman from the rural part of Nepal who was on her 40th day postpartum after she delivered a female baby vaginally at home. The patient had not undergone regular antenatal checkup during pregnancy. On her 5th postpartum day, she had a severe headache and fever that did not subside on taking over-the-counter drugs. During the same period, she developed shortness of breath for 1 day and had palpitation and increased sweating, for which she visited the local community hospital 4 days after the symptom onset where she was examined and found to have bilateral basal crackles, parasternal heave and thrill. Her hemoglobin was 5.2 gm/dl, platelet 69 000/mm^3^, sodium level of 131.6 meq/l, potassium level of 2.4 meq/l and Scrub IgG antibody was positive. ECG revealed T-wave inversion in leads V1–V4. She was diagnosed as a case of postpartum cardiomyopathy with severe anemia with hypokalemia.

Then, the patient was referred to our center on 15th day of postpartum and underwent various investigation (Table 1). IV potassium replenishment was done with 20 mEq of KCL in 1 liter of normal saline over 2–4 hours which brought the serum potassium level to acceptable limits. Two pints of blood was transfused to normalize the hemoglobin level with a furosemide cover. Standard heart failure therapy was initiated which comprised of intravenous (IV)furosemide to relieve pulmonary congestion with concurrent monitoring of the corrected serum potassium levels. Fever and headache were managed with IV analgesics and antipyretics. After the relief of acute symptoms, the patient party opted for discharge because of financial burden, without any further diagnostic interventions. However, after 2 weeks, she again presented on 40th postpartum day with the complain of high-grade fever for 3 days, associated with chills, rigor and sweating. The maximum documented temperature was 103 °F. She also complained of episodic shortness of breath, mostly during exertion, which gradually worsened. Palpitation and orthopnea were also noted. In addition, she had dry cough with no diurnal variation. Similarly, she had a pulsating type of non-radiating fronto-temporal headache which was not associated with nausea, vomiting, photophobia or phonophobia. There was no history of any illicit IV drug abuse, childhood cardiac illness, or recent history of tooth extraction.

**Table 1:** Blood test results.

Blood Parameters	Results
WBC	11 210
RBC	2.75
Hb	7.26
PCV	21.1
MCV	78.18
MCHC	26.55
Platelets	3 25 000
RDW-CV	18.2
Neutrophils	81.0
Lymphocytes	14.0
Monocytes	04.0
Eosinophils	01.0
Basophils	0.0
ESR	40
Procalcitonin	2.44
NT-Pro BNP	1740.000
Iron	29.2
TIBC	300
Ferritin	376.0

HIGHLIGHTS

- Isolated pulmonary valve endocarditis in a postpartum woman without any conventional risk factors such as intravenous drug use, cardiac devices, or structural heart disease.
- Initially misdiagnosed as scrub typhus and postpartum cardiomyopathy, delaying definitive treatment for over 5 weeks.
- Severe postpartum anemia (Hb 5.2 g/dl) and transient immune suppression are proposed as novel predisposing factors for right-sided infective endocarditis.
- Pulmonary valve vegetation clearly visualized on transthoracic echocardiography (TTE), demonstrating the diagnostic utility of TTE even for right-sided lesions.
- Successful empirical treatment using Linezolid, Clindamycin, and Rivaroxaban in a culture-negative, resource-limited setting, highlighting therapeutic adaptability where standard options are unavailable.

**Figure 1.**
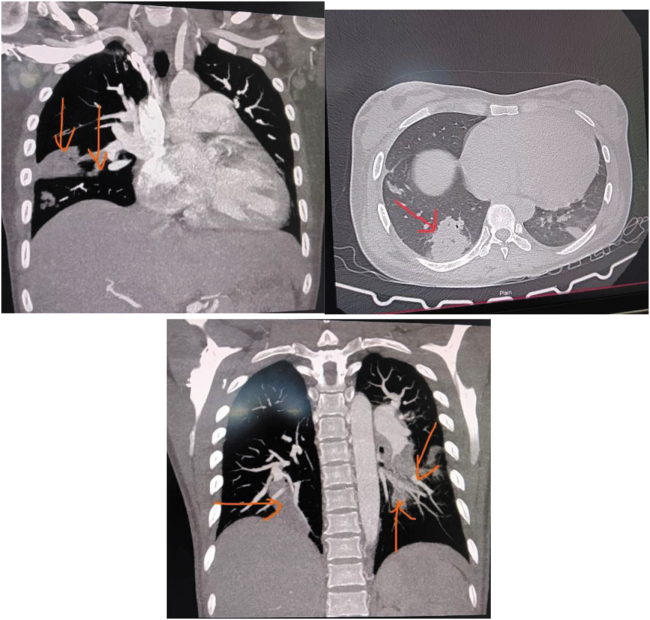
Chest X-ray showing opacification in the mid and lower zones of the right lung, PA view.

**Figure 2.**
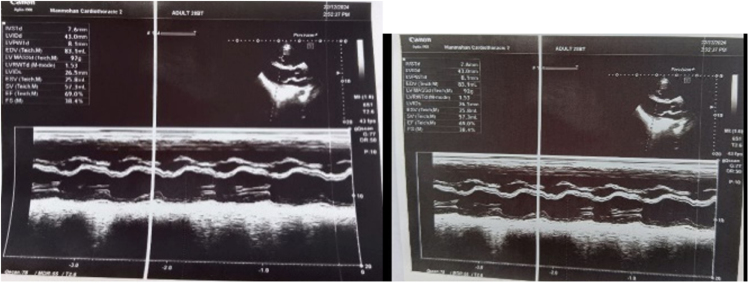
Echocardiographic finding shows hypoechoic oscillating structure of 32 mm × 9 mm attached to the pulmonary valve, with dilated right atrium and right ventricle.

During this visit, she was evaluated for infectious etiology, including *Brucella* antibody test, dengue antibody test, scrub typhus antibody test and *Leptospira* antibody test and all were found to be negative. Further, acid-fast bacilli (AFB) stain for tuberculosis was done which turned out to be negative. Blood culture yielded no significant growth. Gynecological consultation was done for suspicion of endometriosis, but was ruled out due to the absence of vaginal foul-smelling discharge. Blood investigation was done and is given as follows:

Chest X-ray revealed wedge shaped consolidation in mid and lower zones of the right lung as shown in Figure 1. Given the prolonged history of fever and shortness of breath with palpitation and orthopnea, echocardiography was advised. It revealed hypo-echoic oscillating structure of 32 mm × 9 mm attached to the pulmonary valve (probably clot). Also, there was severe pulmonary regurgitation with mild eccentric tricuspid regurgitation. Right atrium and ventricles were dilated as shown in Figure 2. Oscillating structure in the pulmonary valve warranted the need of CECT Pulmonary Angiography to rule out possible embolism via pulmonary artery. Wedge shaped consolidation in the middle and lower lobe of right lung further provided us with a benefit of doubt to undergo pulmonary angiography.

**Figure 3.**
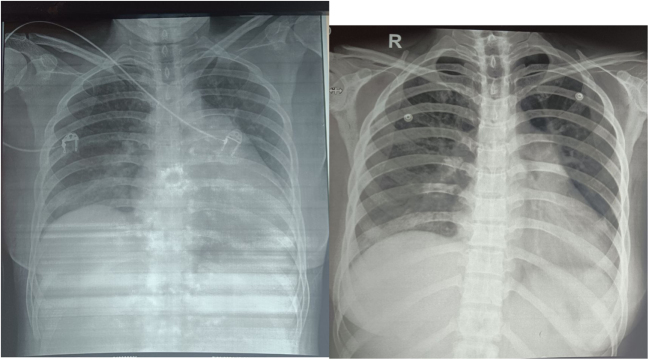
CECT showing hypodense filling defect in the right lower lobe pulmonary artery and its distal branches, subsegmental branches of lateral basal segment of right and middle lobe and the posterobasal segment pulmonary artery, wedge-shaped area of consolidation in right lower lobe, right middle lobe and left lower lobe shown by the red-colored arrow.

She was advised for Contrast Enhanced CT (CECT) of the chest whose findings are given in the Figure 3. It showed hypodense filling defect in the right lower lobe pulmonary artery and its distal branches, subsegmental branches of lateral basal segment of right and middle lobe and postero-basal segment of the pulmonary artery. This was suggestive of acute pulmonary thromboembolism with pulmonary infarcts since, a technically adequate CTPA is sufficient and considered gold standard criterion for diagnosing pulmonary embolism. Likewise, in accordance with the Duke’s criteria for infective endocarditis, a major criterion of evidence of endocardial involvement and minor criteria of fever and septic pulmonary infarct have been met. Although adequate requirements for definitive diagnosis of infective endocarditis are not present, the clinical and imaging evidence is overwhelming. So, the likely diagnosis of “Infective Endocarditis of Pulmonary Valve with septic embolism with Iron deficiency anemia” was made.

Further, 1 pint of blood was transfused. In addition, medications: Tab. Spironolactone 25 mg, Tab. Folic acid, Tab. Clindamycin 600 mg, Tab. Linezolid 600 mg, Pantoprazole, Tab. Lasix 20 mg, and Tab. Rivaroxaban 15 mg was given to the patient. Her condition significantly improved with good response to the above medications. She was advised to follow up in 1 month with CBC, RFT and ECG reports to the cardiology department.

## Discussion

Right-sided infective endocarditis makes up about 5–10% of all endocarditis cases^[^6^]^. It’s commonly linked to intravenous drug use, implanted heart devices, and the use of central venous catheters. Pregnancy and postpartum infective endocarditis (IE) are serious conditions that pose a significant threat to both the mother and the baby, with mortality rates between 10 and 15%^[^7^]^. Most cases involve the left side of the heart, particularly the mitral valve. When it comes to right-sided infective endocarditis (IE), the tricuspid valve is almost always involved – over 90% of the time but the infection spreads to nearby structures like the pulmonary valve, the right ventricular outflow tract, the right ventricular free wall, or even remnants of the right atrium, such as the Eustachian valve^[^6,8^]^. Right-sided infective endocarditis (RSIE) in a postpartum patient without conventional risk factors presents a clinical enigma that challenges established understanding of how such infections arise, are diagnosed, and managed. This case, situated at the intersection of obstetrics, cardiology, and infectious disease, necessitates examining the interplay between postpartum physiological vulnerability, sociocultural health determinants, and diagnostic challenges.

The postpartum period is a temporary state of weakened immunity, driven by hormonal shifts, physical stress, and delayed recovery of the uterus. Progesterone’s lingering suppression of the immune system, meant to protect the baby during pregnancy, can leave the body more vulnerable to infections for weeks after delivery^[^9^]^. While the pulmonary valve is rarely affected by infective endocarditis (5–10% of cases), it can become a site of infection if the blood vessel lining is damaged. The increased blood flow and volume during pregnancy can cause minor damage to heart valve linings, creating a surface where bacteria can attach^[^10^]^. While left-sided valves are more commonly affected, right-sided involvement here suggests a brief bacterial infection in the bloodstream from an unclear source. Severe anemia, with hemoglobin as low as 5.2 g/dl, likely worsened the immune system’s ability to function properly^[^11^]^. Iron is essential for neutrophils to work effectively and generate the oxidative burst needed to kill bacteria. A lack of iron weakens this process, making it harder for the body to clear infections and allowing them to persist^[^11^]^. The postpartum period might be an overlooked time of increased vulnerability for unusual cases of infective endocarditis (IE). This means healthcare providers should be extra cautious with postpartum patients who develop a fever, even if they do not have the usual risk factors for IE.

This case underscores the risks of anchoring bias, particularly in resource-limited settings. Initially, the overlapping symptoms such as shortness of breath, difficulty breathing while lying flat, and palpitation led to a misdiagnosis of postpartum cardiomyopathy, a condition with similar clinical features. It was only through echocardiography and CECT that the true diagnosis –RSIE was identified, revealing the presence of pulmonary valve vegetation. This emphasizes the need for a thorough and open-minded approach, especially when dealing with rare or atypical presentations.

While transthoracic echocardiography (TTE) is the cornerstone of IE diagnosis, its sensitivity for right-sided vegetation is suboptimal, ranging between 50 and 70%^[^6^]^. In this case, the pulmonary valve’s anterior position likely enhanced detection, but reliance on TTE alone in low-resource regions risks underdiagnosis. Despite negative blood cultures, empirical therapy succeeded, suggesting fastidious organisms (e.g., *Bartonella, Coxiella*) or prior antibiotic use^[^12^]^. In rural Nepal, pre-hospital antibiotic misuse is rampant, often obscuring microbiological evidence. Transient bacteremia from hidden infections like subclinical endometritis or pelvic infections often seen after home deliveries, could have been the source of the pulmonary valve infection. Even though no obvious genital infection was noted, hard-to-detect pathogens such as *Streptococcus agalactiae* (Group B Streptococcus), a known postpartum risk, might have played a role, especially in cases where standard cultures fail to identify the cause^[^13^]^. The use of clindamycin and linezolid, instead of standard bactericidal drugs like β-lactams or vancomycin, likely reflects challenges in high-MRSA (Methicillin Resistant *Staphylococcus aureus*) regions, offering broad gram-positive and anaerobic coverage despite limited IE evidence. Rivaroxaban’s role in IE is debated due to bleeding risks but it may be justified for septic pulmonary emboli, with early studies suggesting it reduces clot burden in right-sided IE^[^14^]^.

The patient’s home delivery, lack of antenatal care, and delayed presentation reveal systemic gaps in rural maternal healthcare where births occur at home, often without skilled help. Unsterile practices or perineal trauma could introduce pathogens, causing transient bacteremia. Fever was initially misattributed to anemia or scrub typhus (positive IgG), delaying advanced imaging. In low-resource settings, febrile illnesses are often managed symptomatically, overlooking rare diagnoses like IE. This case calls for integrating IE screening into postpartum fever protocols, especially in regions with high home birth rates. Moreover, postpartum state might be a fruitful addition to the predisposing factors in the minor criterion of Modified Duke’s Criteria for diagnosis of IE but it requires further evidence.

This case redefines the risk profile of RSIE, identifying the postpartum period as a potential risk factor. It calls for routine echocardiography in postpartum patients with relapsing fever, even without typical risk factors. Additionally, training community health workers in sterile delivery practices and early sepsis recognition is crucial.

## Conclusion

This case highlights postpartum infective endocarditis as a potential complication of pregnancy-related physiological changes, even in patients without traditional risk factors. The interplay of progesterone-induced immunosuppression, hemodynamic stress, and severe anemia likely contributed to this rare presentation of pulmonary valve endocarditis. Our findings emphasize the importance of considering endocarditis in postpartum patients with persistent fever and the need for heightened clinical suspicion in resource-limited settings where diagnostic challenges may delay identification. This case underscores the necessity of improved maternal healthcare monitoring and sterile delivery practices to prevent such serious infectious complications.

## Data Availability

Data are available upon reasonable request.
